# Off‐axis dose distribution with stand‐in and stand‐off configurations for superficial radiotherapy treatments

**DOI:** 10.1002/acm2.12730

**Published:** 2019-10-12

**Authors:** Keith Davey, Margaret Moore, Sinéad Cleary, Christoph Kleefeld, Mark J. Foley

**Affiliations:** ^1^ School of Physics National University of Ireland Galway Galway Ireland; ^2^ Radiotherapy Department University Hospital Galway Galway Ireland

**Keywords:** off‐axis dose distribution, stand‐in, stand‐off, superficial radiotherapy

## Abstract

Current practice when delivering dose for superficial skin radiotherapy is to adjust the monitor units so that the prescribed dose is delivered to the central axis of the superficial unit applicator. Variations of source‐to‐surface distance due to patient’s anatomy protruding into the applicator or extending away from the applicator require adjustments to the monitor units using the inverse square law. Off‐axis dose distribution varies significantly from the central axis dose and is not currently being quantified. The dose falloff at the periphery of the field is not symmetrical in the anode–cathode axis due to the heel effect. This study was conducted to quantify the variation of dose across the surface being treated and model a simple geometric shape to estimate a patient’s surface with stand‐in and stand‐off. Isodose plots and color‐coded dose distribution maps were produced from scans of GAFChromic EBT‐3 film irradiated by a Gulmay D3300 orthovoltage x‐ray therapy system. It was clear that larger applicators show a greater dose falloff toward the periphery than smaller applicators. Larger applicators were found to have a lower percentage of points above 90% of central axis dose (SA90). Current clinical practice does not take this field variation into account. Stand‐in can result in significant dose falloff off‐axis depending on the depth and width of the protrusion, while stand‐off can result in a flatter field due to the high‐dose region near the central axis being further from the source than the peripheral regions. The central axis also received a 7% increased or decreased dose for stand‐in or stand‐off, respectively.

## 1 INTRODUCTION

Superficial skin radiotherapy treatments are often carried out utilizing superficial photons in the kilovoltage range from 50 to 150 kVp,^1^ where dose is calculated on the surface of the central axis. This ignores dose toward the edges of the field, where dose decreases due to the method of production of superficial photons. Superficial therapy units produce photons using an x‐ray tube, similar to that used for the production of diagnostic images such as radiographs or computed tomography (CT) images.^2^ The electron emitted by the cathode does not interact at the surface of the anode. Instead, the electrons travel a depth inside the anode material and the photons produced must traverse the anode to reach the patient’s surface. This leads to a reduction of intensity of the beam at the edges of the field in‐plane, or perpendicular to the anode–cathode axis. A similar reduction occurs in the anode–cathode axis, but the reduction is asymmetrical due to the heel effect. Therefore, there is variation in dose distribution across the treatment field which should be quantified.

**Figure 1:**
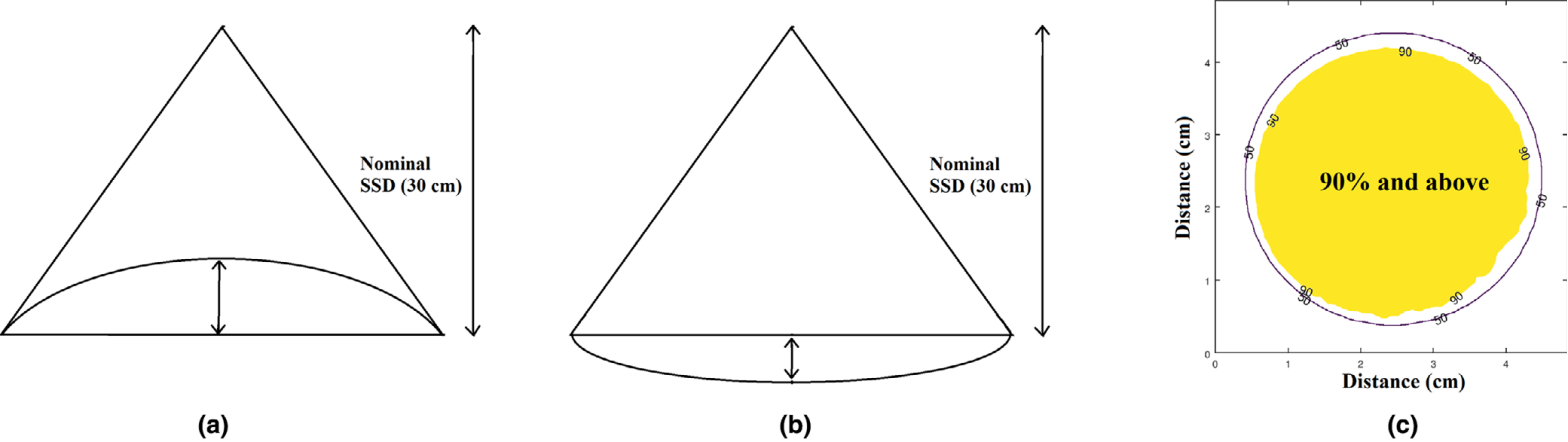
Schematic depicts (a) stand‐in configuration where the patient surface protrudes into the applicator, (b) stand‐off where the patient surface is removed from the applicator surface, and (c) the surface area of 90% (SA90) dose parameter.

Stand‐in occurs when some part of a patient’s anatomy protrudes inside an open‐ended applicator. Stand‐off is a situation where the shape of the patient’s anatomy results in a cavity between the reference plane at the end of the applicator and the patient’s surface. Surface area of 90% (SA90) dose is a parameter defined in this study to describe the percentage of surface area inside an applicator which exceeds 90% of the dose at the central axis. This is useful as it allows a sense of what proportion of the treatment field is being underdosed. Figure 1 displays the patient geometry and surface area dose parameter graphically.

Previous studies^3^, ^4^, ^5^, ^6^, ^7^, ^8^ have almost exclusively focused on dose variation with stand‐off at the central axis. While this shows that dose varies with stand‐off, it neglects the additional dose variation off‐axis which is compounded by the loss of dose due to increased source to surface distance (SSD). Also, many studies such as Gerig et al.^3^ and Gräfe et al.^4^ only investigated applicators which featured a plastic cap at the applicator end; thus, no stand‐in investigation was possible. Gräfe et al.^4^ found no clinically significant variation of dose from the ISL up to 10 cm stand‐off with their investigated beam energies and applicators. Closed‐ended applicators of dimensions 4 × 4, 10 × 10, and 20 × 20 cm^2^ were investigated at beam energies of 100, 150, and 200 kVp. Clinically significant variations began to first occur at 1.8 cm stand‐off for the 4 × 4 cm^2^ applicator at 150 kVp.

The applicators used in this study featured open ends for the investigated energies of 100 and 140 kVp enabling protrusion into the applicator. Li et al.^5^ and Aspradakis and Zucchetti^6^ investigated both open‐ended and closed‐ended applicators but did not investigate stand‐in. Li et al.^5^ used two open‐ended applicators, a 4 cm circular cone and an 8 × 8 cm^2^ square cone, at 100 and 300 kVp. The closed‐ended applicators were square cones of 10 × 10, 12 × 12, and 20 × 20 cm^2^. Dose was measured at certain stand‐off distances for each energy and applicator combination. Their findings suggest that open‐ended applicators show no clinically significant variation from dose calculated by adjusting for the inverse square law (ISL) up to 15 cm stand‐off. For closed‐ended applicators, exceeding 1 cm stand‐off at 100 kVp results in clinically significant dose variation from that calculated with the ISL. Here, clinically significant is deemed as variance exceeding 5%, as stated by the International Commission of Radiation Units and Measurements.^9^

Evans et al.^7^ utilized a Gulmay D3300 Superficial Skin Therapy Unit (Gulmay Medical, Camberley, UK) using both open‐ and closed‐ended applicators at four beam energies. Contrary to Li et al.^5^ and Gräfe et al.^4^, Evans et al.^7^ concluded that there was no clinically significant variation of measured dose at the central axis from that calculated when the ISL was considered. This is potentially due to their use of a Farmer chamber instead of the Markus chamber used by Gräfe et al.^4^ which has a significantly smaller sensitive volume of 0.055 cm^2^ compared to 0.22 cm^2^ for the Farmer chamber. Evans et al.^7^ investigated beam energies of 70 and 100 kVp with open‐ended applicators with diameters of 2.5, 5, and 10 cm. Beam energies of 180 and 250 kVp were investigated using closed‐ended applicators of 4 × 4, 10 × 12, and 20 × 20 cm^2^.

In vivo measurements were taken by Palmer et al.^8^ using micro silica beads as thermoluminescent detectors (TLDs). They found that in half of the patient treatments investigated, off‐axis dose exceeded 5% variance relative to the central axis dose. To the best of the author’s knowledge, this study is the only previous study in the literature that has any investigation of dose off‐axis, highlighting the need for investigation into dose off‐axis relative to the central axis in kilovoltage radiotherapy. This is the closest study found in the literature to the investigation undertaken in this paper. This study aims to quantify the variability of dose off‐axis and produce isodose plots similar to those produced for megavoltage photons and electrons. This dose quantification will further knowledge of superficial treatment fields in order to determine if adjustments to current treatment planning practice are required for superficial treatments.

## 2 MATERIALS & METHODS

### 2.1 Superficial treatment unit

The treatment unit used in this study was a Gulmay D3300 orthovoltage x‐ray therapy system which can produce beam energies from 40 to 300 kVp. Two beam energies, 100 and 140 kVp, and seven applicators ranging from a 2‐cm circle to a 10 × 10 cm^2^ are used clinically in the Radiotherapy Department of University Hospital Galway. The seven applicators used at the above beam energies are open ended. All combinations of the two beam energies and seven applicators were utilized for flat field measurements.

### 2.2 Film calibration

Before performing measurements, it is necessary to calibrate the GAFChromic EBT‐3 film^10^ at the kilovoltage beam energies in use. It was decided to calibrate at 100 kV only as previous studies established that the energy dependence of GAFChromic EBT‐3 film between 100 and 140 kVp was negligible.^11^, ^12^ The calibration required a range of dose values which was achieved by irradiating from 10 to 1000 MU. The absolute dose delivered was then calculated by multiplying the delivered dose by the applicator factor determined during commissioning. The calibration procedure described by the vendor of the FilmQA Pro^10^ software was followed (http://www.gafchromic.com/documents/Efficient%20Protocols%20for%20Calibration%20and%20Dosimetry.pdf). Twenty‐four hours between irradiation and scanning using an Epson 10000 XL scanner was required. It is important to keep the orientation and position of the film constant throughout all scanning procedures to negate any longitudinal variance in the scanner itself. Any settings that would process the image were disabled, such as Unsharp Mask and Color Restoration. The film was scanned with the 48‐bit color mode and with 200 dpi in the TIFF format. Known dose was calculated by multiplying the delivered monitor units by an applicator correction factor documented during commissioning of the unit.

**Figure 2:**
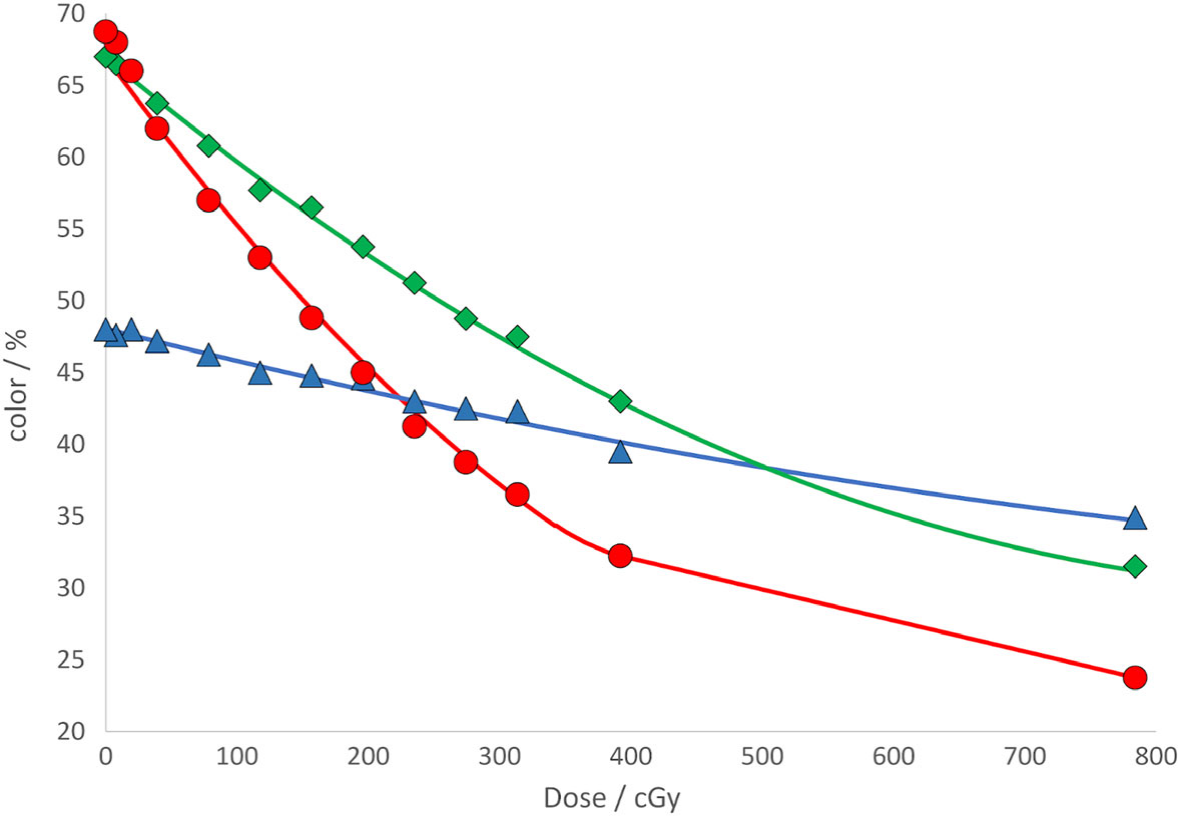
Calibration data produced by FilmQA Pro.

Next, the scanned film was imported into the FilmQA Pro^10^ software as a “Film Calibration (Ordinary)” file. The exposed regions and an unexposed region were selected as regions of interest and a dose value was assigned to each region. This produced a calibration curve in the red, green, and blue channels. A graph produced with this calibration data is shown in Fig. 2. This file was then saved in FilmQA Pro and applied as a calibration for all the film measurements.

### 2.3 Surface and depth measurements

Measurements with the GAFChromic EBT‐3 film were taken on the surface of 10 cm of backscatter material as well as at 5 and 10 mm depth underneath Bart’s water tissue equivalent plastic (Barts Health NHS Trust, Clinical Physics CSS CAG, The Royal London Hospital, UK). The applicator end was placed on top of the film and backscatter material, and where necessary, shims were used to eliminate any air gaps between the applicator end and the film. The film pieces were cut such that each piece was 1.5–2 cm longer and wider in each direction than the field size being measured. Each film was exposed to 200 MUs due to the linearity of the response between 150 and 250 cGy. Measurements were taken at the 100 and 140 kVp beam energies, and thus, a total of six sets of measurements were taken. Each applicator, energy, and depth combination film were carefully scanned following the procedure described above and imported in the FilmQA Pro software as “Dose Map (single scan)” files. A square region of interest was selected around the irradiated area, and the dimensions of the selected region noted. The dimensions are important to note to enable conversion of the scale into millimeters. The “Surface Plot” option was selected on the right‐hand side of the FilmQA Pro window and the plot data imported into a spreadsheet. It is important to note that FilmQA Pro exports the data as a 51 by 51 grid of points within the region of interest. This has the effect of restricting the spatial resolution depending on the field size. That is, larger field sizes will have a worse spatial resolution than smaller field sizes. An Octave script (GNU Octave, version 4.2.2) was written by the author to extract the data from the spreadsheet and to perform analysis to produce isodose plots and color‐coded dose distribution maps. For all applicators, every dose point inside the applicator was compared to the dose at the central axis. The applicator size was approximated by selecting points above 50% of the central axis dose, which gave appropriate shapes and sizes for each applicator. The SA90 was calculated by finding the number of data points above 90% of the central axis dose and calculating the percentage of those points compared to the total.

Measurements were also taken using a PTW LA‐48 Linear Chamber Array (PTW GmbH, Freiburg, Germany), which consists of 47 liquid‐filled chambers containing isooctane. The intention of these measurements was to independently verify that the GAFChromic EBT‐3 film is suitable for use with kilovoltage photons. The GAFChromic EBT‐3 film is already backed up in the literature as suitable for use with kilovoltage photons^13^, ^14^, ^15^, ^16^. The PTW LA‐48 array measurements were taken under 5 mm of liquid water in a PTW MP3 Water Tank (PTW GmbH, Freiburg, Germany), allowing a 1‐mm spatial resolution despite the 8 mm spacing between each chamber of the array. The measurements were taken through the central axis in the cross‐ and in‐plane directions and compared to similar plots taken from the GAFChromic EBT‐3 film data.

### 2.4 Stand‐in and stand‐off measurement and simulation

**Figure 3:**
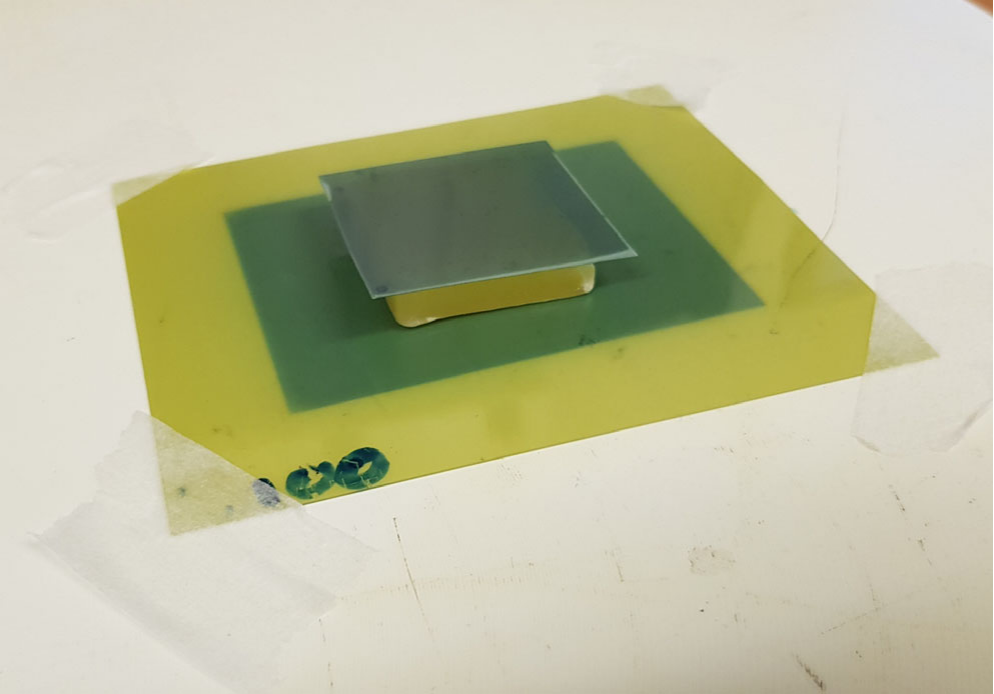
Setup of stand‐in measurements with film underneath and on top of wax block.

**Figure 4:**
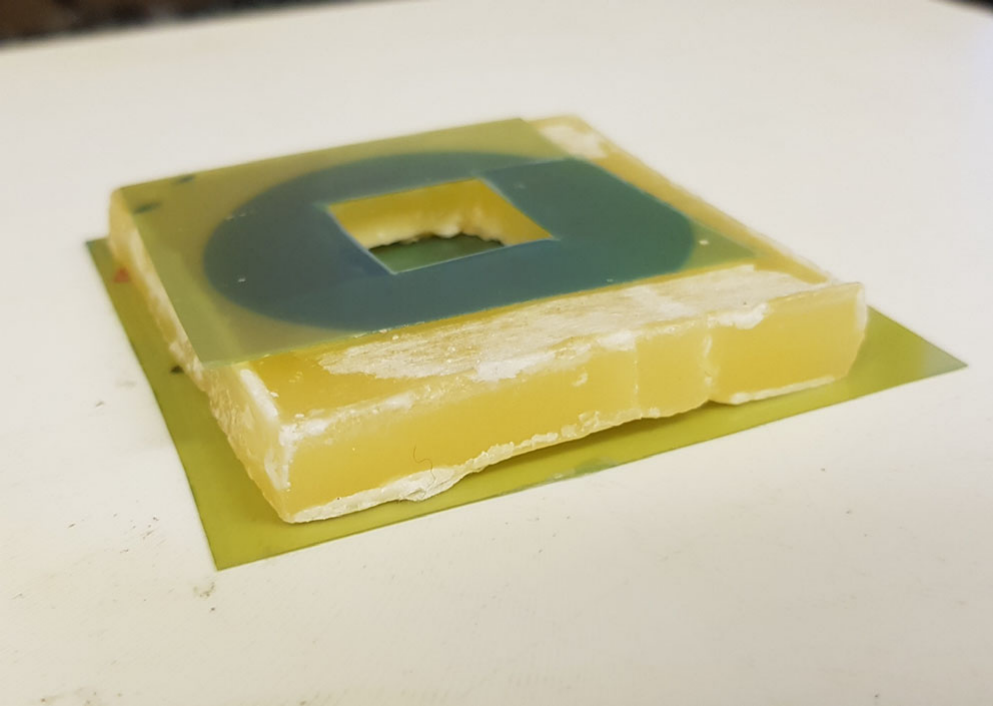
Set‐up of stand‐off measurements with film underneath block and on top in order to measure dose at bottom of cavity and at the reference plane.

To measure simulated situations of stand‐in, wax was formed into a square block of approximately 8 mm thickness. Film was placed both underneath and on top of the block (Fig. 3). For stand‐off simulations, a square hole was cut into a block of wax of the same thickness and film was placed both in the cavity and on the surface of the wax block (Fig. 4). Effectively, this means that the resulting isodose plots displayed dose on the surface as opposed to specified distances from the applicator end. The different wax blocks were measured, and their dimensions used to apply ISL corrections to the measured dose for a flat surface. Thus, dose on top of the wax block for stand‐in or inside the cavity for stand‐off could be simulated. This was done using a GNU Octave script which applied the appropriate dose correction factor due to the ISL to the flat surface measurement data. The efficacy of the model was verified by comparing the calculated values from the model to the measured values.

### 2.5 Case simulation

A GNU Octave script was written which created a geometric shape in the form of a Gaussian of adjustable height and thickness and calculated the ISL correction factor for each data point. The correction was applied to the data from flat surface measurements and isodose plots and dose distribution maps created to highlight the variation of dose with a shape replicating the nonuniformity of the surface of a patient. The aim was to discover if the effect of a nonuniformity on a patient could be modeled by taking the inverse square law into account for each data point. By inverting the direction of the Gaussian, stand‐off was also simulated.

## 3 RESULTS & DISCUSSION

### 3.1 Surface measurements

**Figure 5:**
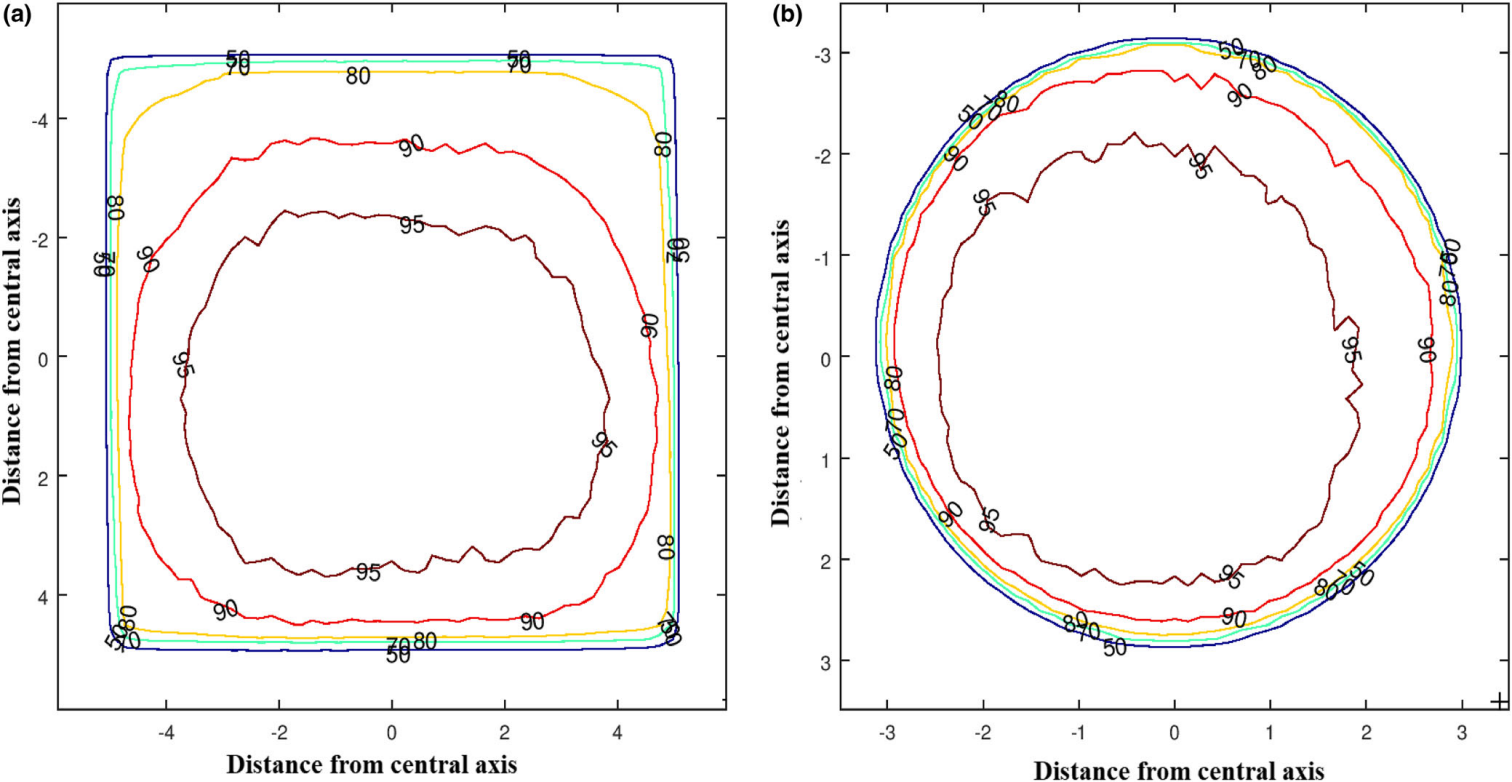
Isodose profiles for the (a) 10 × 10 cm^2^ and (b) 6 cm applicators at 100 kVp.

**Figure 6:**
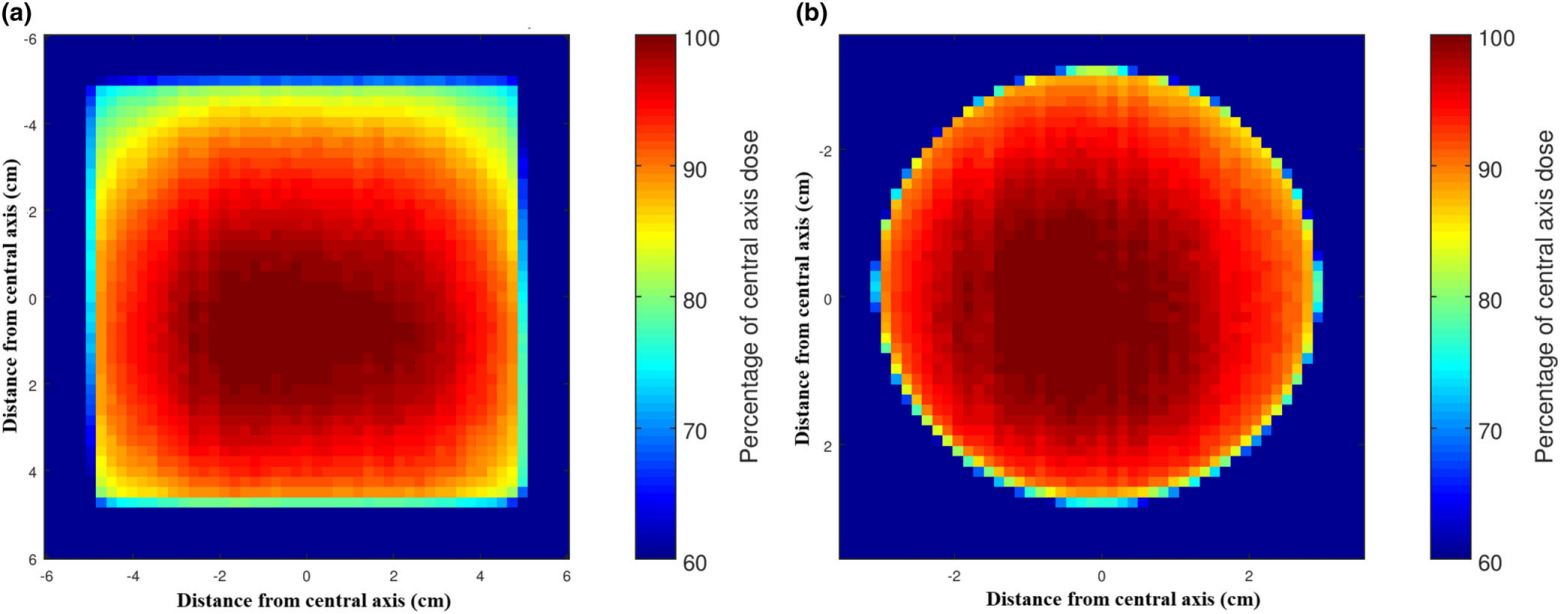
Color‐coded dose distribution maps for the (a) 10 × 10 cm^2^ and (b) 6 cm applicators at 100 kVp.

**Figure 7:**
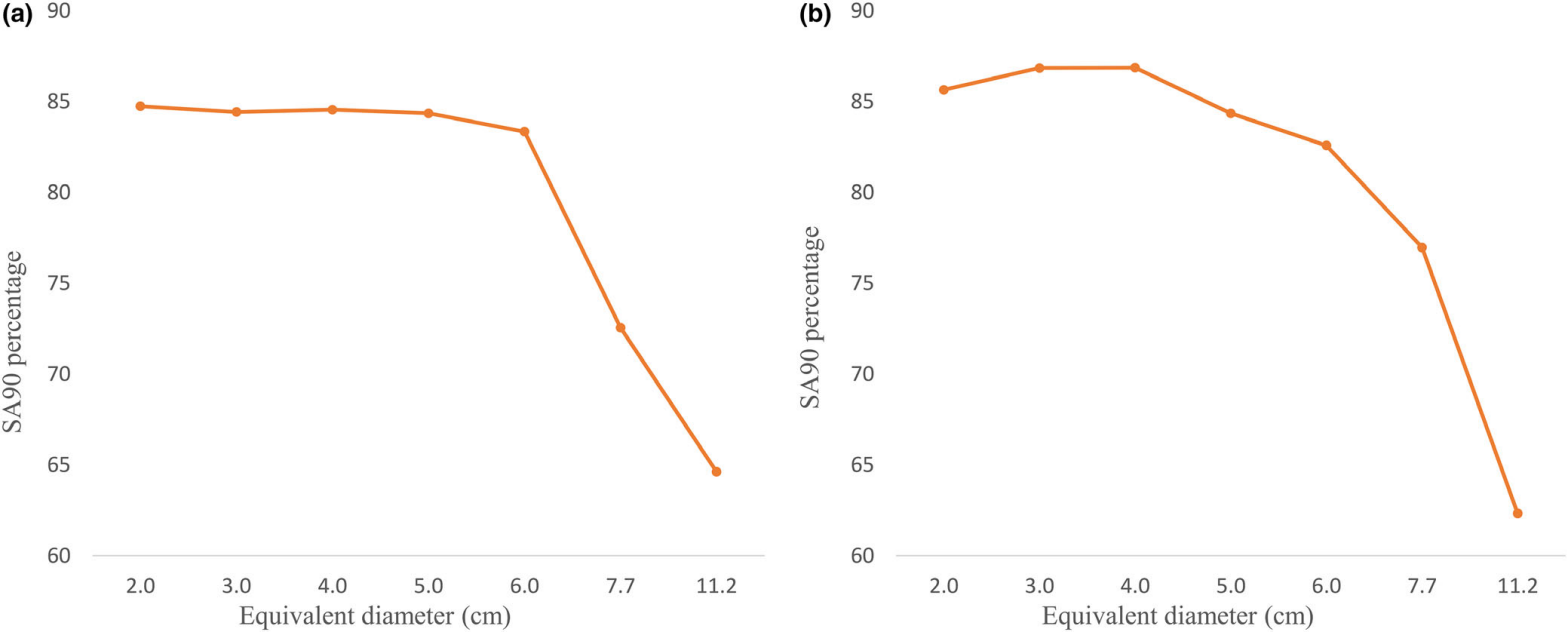
SA90 for each applicator at (a) 100 kVp and (b) 140 kVp beam energies.

Figure 5 displays the isodose plots produced for the 10 × 10 cm^2^ applicator and the 6 cm circular applicator at 100 kVp beam energy. For the same two cases, color‐coded dose distribution maps were produced (Fig. 6). This highlights the variation in dose across the field even for flat surfaces. Figure 7 shows that the SA90 decreases with increasing applicator size for the 100 and 140 kVp beam energies, respectively. As the imported data from the scans were in the form of a square grid, circular fields were selected by comparing data points inside a circle with a radius equal to the distance to the 50% dose values. For the square and rectangular fields, a similar approach was taken but with appropriate shapes for the field selected.

The isodose plots and dose distribution maps produced provide important visual aids for physicists and clinicians alike in order to be fully informed for patient treatments. There is very limited literature on off‐axis dose distribution for kilovoltage radiotherapy. Palmer et al.^8^ investigated dose off‐axis but not to the extent of this paper. This work builds on previous studies, such as Palmer et al.,^8^ who measured off‐axis dose using TLDs attached to a string which were placed on a patient’s skin during treatments. These plots highlight the need to be aware of field variations to ensure patients are not underdosed.

**Figure 8:**
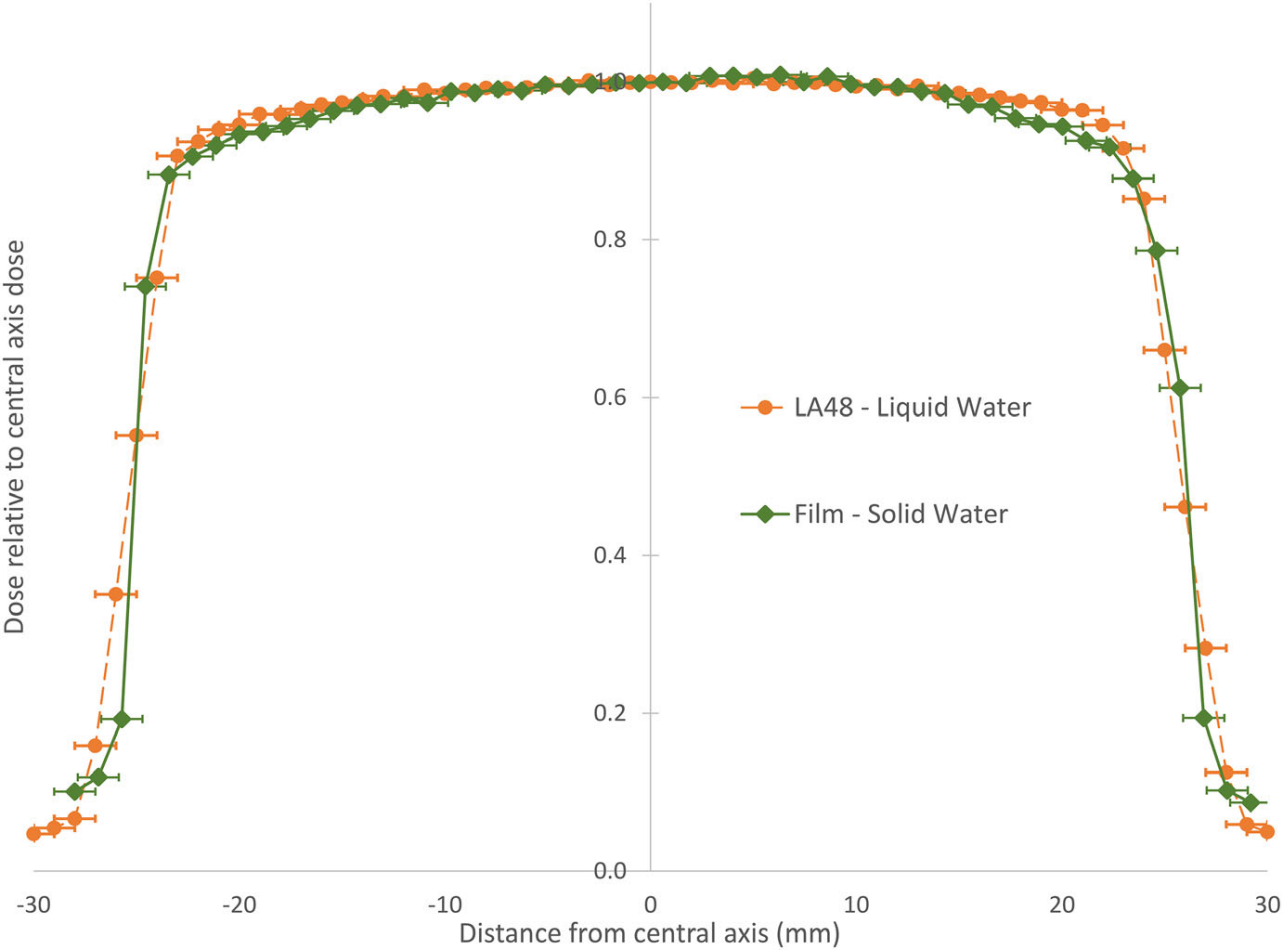
Comparison plot of GAFChromic EBT‐3 film under 5 mm of Bart’s water tissue equivalent material (green diamond) with PTW LA‐48 Array measurements under 5 mm of liquid water (orange circle) at 100 kVp using 5 cm circle applicator.

The PTW LA‐48 linear chamber array measurements were taken through the central axis in the cross‐plane and in‐plane directions. Comparisons with GAFChromic EBT‐3 film measurements in those planes were in good agreement, where the average percentage difference between array and film data points for every applicator were −0.2 ± 0.7% and −1.0 ± 0.4% for the 100 and 140 kVp beam energies, respectively. The results for each beam energy and applicator size are displayed in Table 1. An example of the comparisons between the PTW LA‐48 array and the GAFChromic EBT‐3 film measurements through the central axis in the cross plane is shown in Fig. 8.

**Table 1:** Percentage difference in relative dose for GAFChromic EBT‐3 Film under 5 millimeters of solid water and PTW LA‐48 Linear Chamber Array under 5 millimeters of liquid water.

Applicator size (cm)	Percentage difference, with standard error	
	100 kVp	140 kVp
10 × 10	1.5 ± 0.5%	0.2 ± 0.8%
6 × 8	−1 ± 2%	−2 ± 1%
6 φ	0 ± 1%	−2 ± 1%
5 φ	−1 ± 2%	−2 ± 1%
4 φ	−1 ± 2%	−1 ± 1%
3 φ	−2 ± 2%	−1 ± 2%
2 φ	−1 ± 3%	2 ± 3%

The comparison of the GAFChromic EBT‐3 film measurements to the PTW LA‐48 Linear Chamber Array measurements in Table 1 shows that there is general agreement between the two, confirming the use of the GAFChromic EBT‐3 film for use with the beam energies in question. The largest percentage difference between the GAFChromic EBT‐3 Film and PTW LA‐48 Linear Chamber Array measurements was 2%, with standard errors ranging from 1% to 3%. Overall, Table 1 shows that there is generally agreement between the GAFChromic EBT‐3 Film and PTW LA‐48 Linear Chamber Array measurements. Figure 8 displays the in‐plane comparison of the GAFChromic EBT‐3 film measurements under 5 mm of Bart’s solid water against the PTW LA‐48 Linear Chamber Array measurements under 5 mm of liquid water. A 1 mm uncertainty in detector placement relative to the orthovoltage unit has been included in the graph. Similar trends were found for all other applicators investigated at both 100 and 140 kVp. Any differences between the two measurements could be explained by the difficulty with reproducible setup on the Gulmay D3300 orthovoltage unit. The use of shims was required in order to remove air gaps between the applicator end and the film. Any slight discrepancy between the properties of the solid water used for the film measurements and the liquid water could affect the results. Rippling of the water due to the array’s movements could also affect the results. Finally, there is a metal support bar underneath the PTW LA‐48 Array when used in the PTW MP3 Water Tank which may be producing scatter contributions to dose and affecting the measurements.

Figure 7 shows that the SA90 decreases with increasing applicator size. Figure 6 confirms this, where the smaller SA90 on the larger applicator is visible. This highlights the need to be aware of the variation of the field when planning on patient treatments. Even for the smallest applicator of 2 cm diameter, only 84.8% and 85.7% of the data points were clinically usable for the 100 and 140 kVp beam energies, respectively. This means that approximately 15% of the area within the applicator that is assumed to be receiving the prescribed dose is being underdosed. Considering these results are for a flat surface, a patient may be receiving a clinically usable dose to an even smaller area for a varying patient’s surface. With this information, there are a few changes to practice which could alleviate the dose drop‐off at the periphery. One option is increasing the delivered MUs. This has the benefit of increasing the SA90 but at the expense of increasing central axis dose. Figure 7 shows that this is most feasible for circular applicators and at the 100 kVp beam energy in particular, as the SA90 does not decrease significantly with increasing circular field size at that beam energy. Another option is to use a larger applicator if there is a concern that disease could extend into the periphery of the field where the dose decreases. This method increases coverage but will increase the amount of healthy tissue being irradiated.

### 3.2 Wax stand‐in and stand‐off

**Figure 9:**
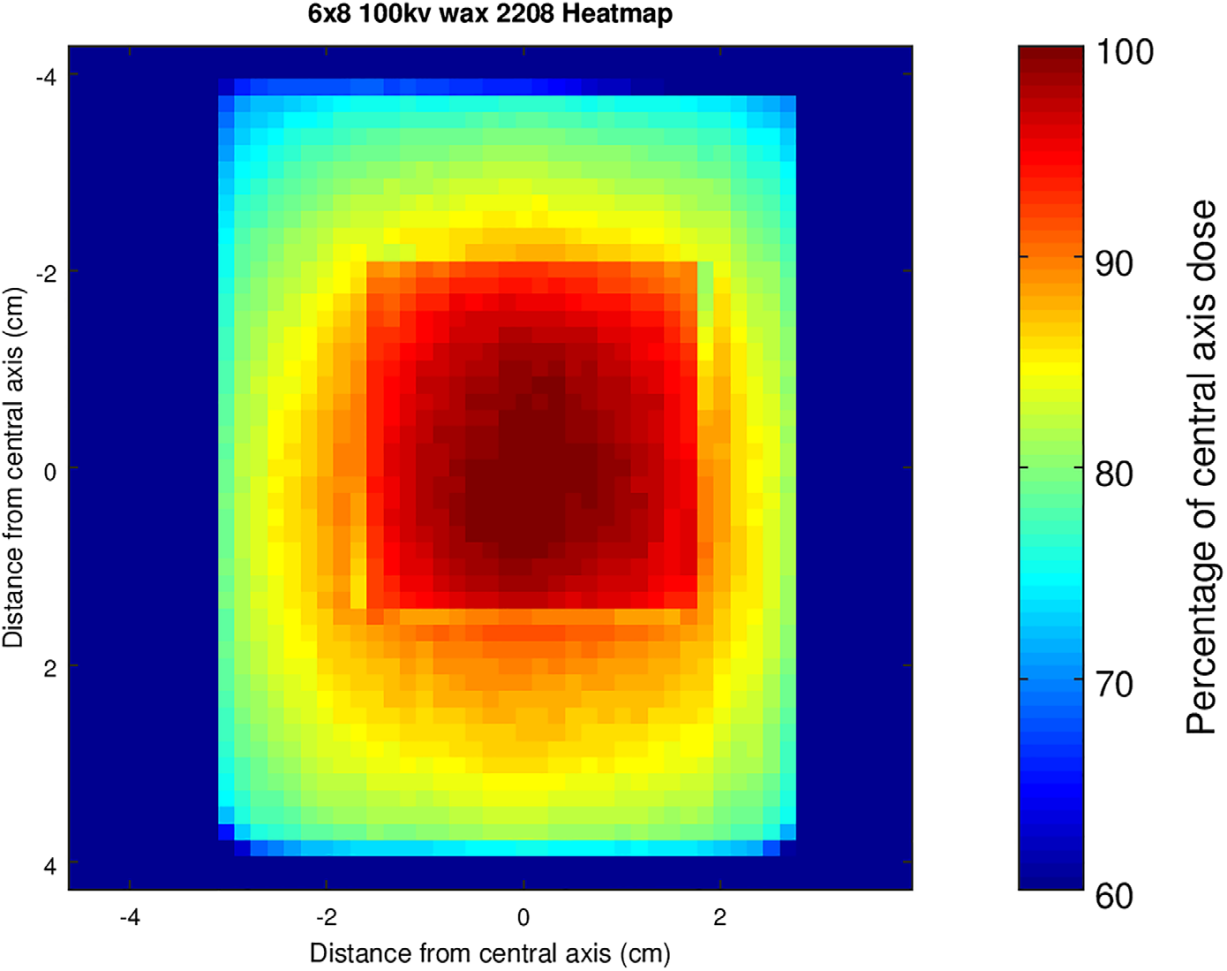
Dose distribution map of measured stand‐in case for 6 cm × 8 cm applicator at 100 kVp beam energy.

The measured stand‐in shows that when the dose across the surface is made relative to the central axis which has a reduced SSD, the periphery of the field receives a significantly reduced dose, dropping to around 75% of the prescribed dose (Fig. 9). To compare calculated dose from the flat surface data to the measured stand‐in data, the datasets had to be matched to the correct coordinates and positioning for accurate comparisons using GNU Octave. The location of the wax block was then determined by subtracting the flat surface data from the stand‐in data, and the coordinates of the position of the block were used to apply the inverse square law correction. The calculated and measured data were then compared by finding the average percentage difference between dose at the same coordinates in each case. For the area of stand‐in, the average percentage difference between the dose values was found to be 2 ± 4%.

**Figure 10:**
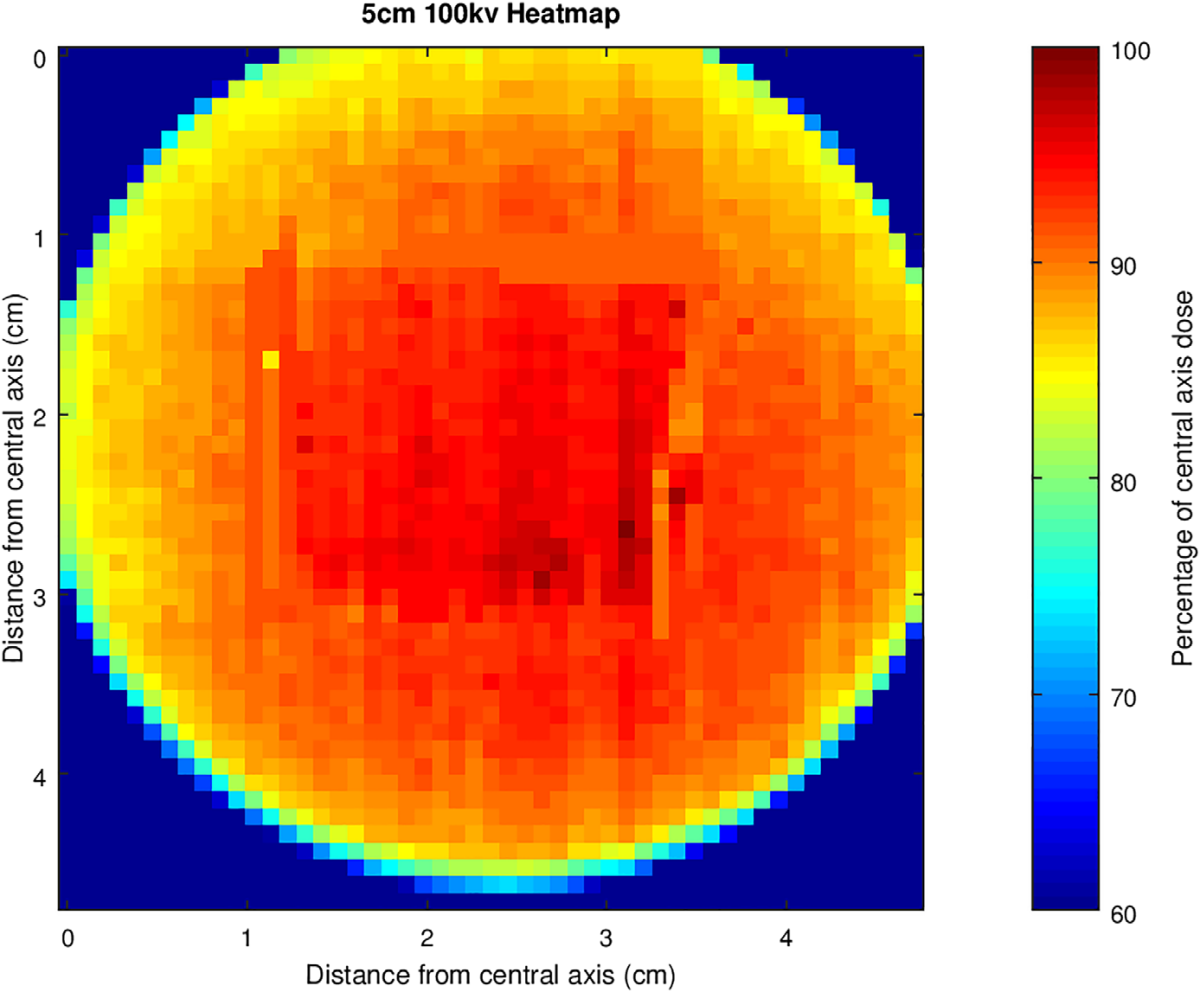
Dose distribution map of measured stand‐off case for 5 cm applicator at 100 kVp beam energy.

**Figure 11:**
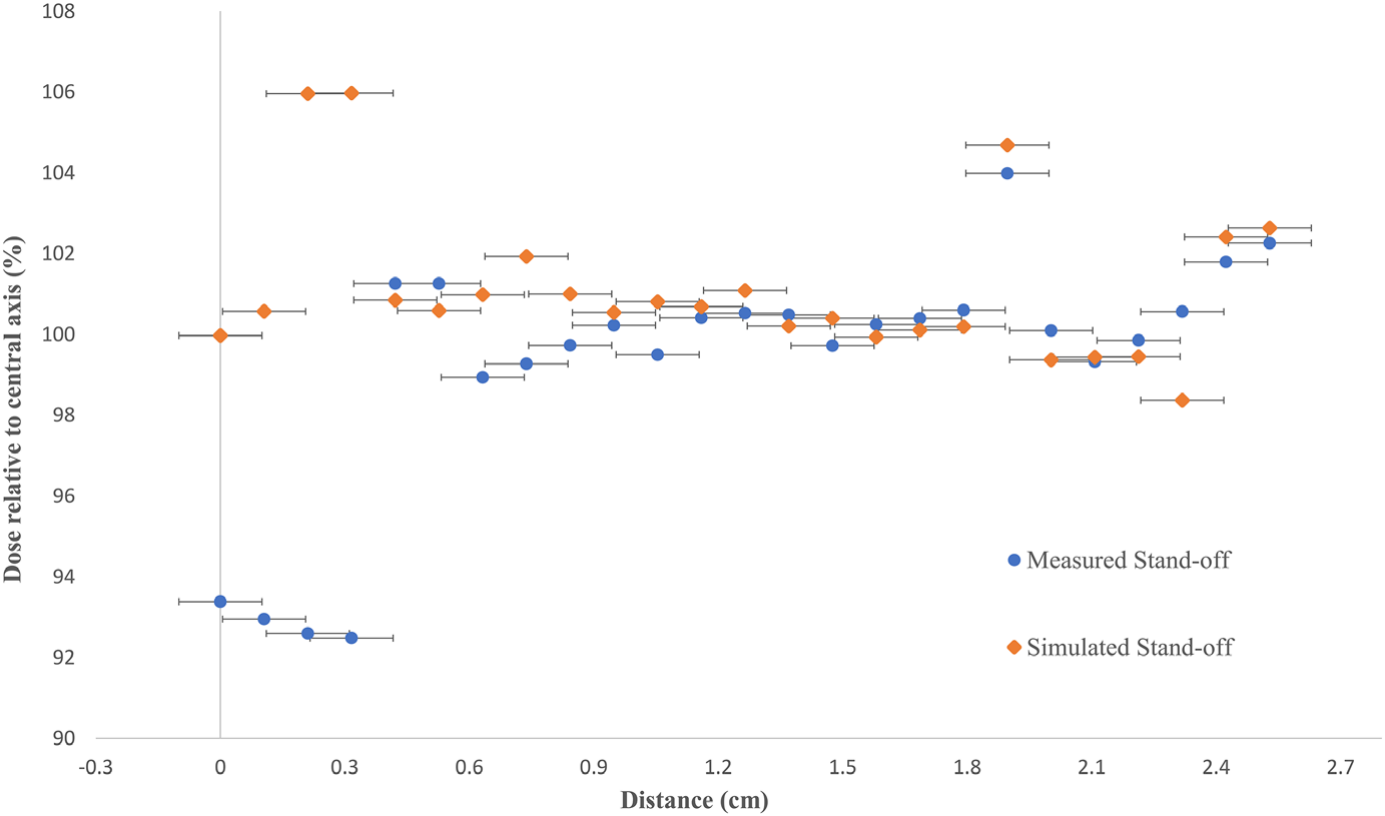
Data through the central axis in the cross‐plane for measured and simulated stand‐off area with 1 mm uncertainty in distance for the reproducibility of the measurements.

The physical measurements of the simulated stand‐off scenario using wax implied that the reduction of dose at the central axis due to increased SSD and the increase in dose relative to the central axis at the periphery due to decreased SSD resulted in a flatter field (Fig. 10). Similar to stand‐in, as the film used for the physical stand‐off measurements and the film used for the flat surface measurements had slightly different scales and positions of the irradiated areas, the GNU Octave script matched the two files so that the correct data points were selected for comparison. The position of the hole in the wax block was determined by subtracting the flat surface measurement from the physical measurement, and then using the coordinates obtained using this method, the ISL was applied to data points on the flat surface measurements corresponding to the location of the hole. Measured and simulated dose values inside the stand‐off area were compared and the average percentage difference between the values was found to be 1 ± 4%. The data for the measured and simulated stand‐off areas through the central axis in the cross‐plane are shown in Fig. 11.

The dose distribution map in Fig. 9 for stand‐in highlights the issue with simply prescribing dose to a patient’s protrusion at the central axis. For the measured case with stand‐in of 8 mm, the dose decreases to 75% at the periphery of the field. Thus, there is a requirement for more knowledge of the variability in dose across a patient’s surface for stand‐in cases. For stand‐off, the dose distribution map shows a significantly flatter field. In this case of 8 mm stand‐off, the raised dose at the periphery relative to the more distant central axis point counteracts the reduction in dose toward the periphery for a flat field. The effect is a flatter field, but for deeper cavities or holes, the central region may end up receiving less dose than the periphery, or for shallower cavities, the pronounced increase of dose at the central axis will reappear.

The results of comparing the stand‐in and stand‐off measurements to calculated dose values from flat surface measurements by utilizing the inverse square law validate the use of the GNU Octave script for the modeling of cases of stand‐in and stand‐off. There was an average percentage difference of 2 ± 4% and 1 ± 4% between the measured and calculated datasets for the stand‐in and stand‐off cases, respectively. Such differences between the values could be explained by error due to the reproducibility of the setup and/or in variations in machine output.

### 3.3 Case simulation

For a 1‐cm stand‐in or stand‐off in the form of a Gaussian profile with full width half maximum of 1.56 cm results in dose to the central axis being 7% higher or lower than the central axis dose for a flat surface. Such a large variation in the central axis alone can result in patients receiving an inappropriate dose. For stand‐in, off‐axis the dose drops off, but remains higher than it would be in a flat surface situation. For stand‐off, moving from the center toward the periphery results in an increased dose due to becoming closer to the anode. This is balanced by the loss of dose moving away from the central axis due to the field variation visible even on flat surfaces.

The use of the model for predicting the dose distribution in stand‐in and stand‐off scenarios was verified by comparing dose calculated using the ISL correction applied to measured flat fields to measured doses using the square wax blocks, where the doses were found to match within 2 ± 4% and 1 ± 4%, respectively. The results from applying a Gaussian to the flat surface data show that it is important to quantify and model the effects the patient’s surface has on the dose distribution. Skin protruding inside the applicator can end up overdosed, and likewise, a cavity between the central axis and the patient’s surface can result in significant underdosing. Both scenarios are clinically significant and should be avoided or quantified.

When the Gaussian was kept to a constant size of height 1 cm and full width half maximum of 1.56 cm, the central region of each of the larger applicators was the only affected region. Even within these smaller regions, the dose reaches 107% and 93% of central axis dose on flat surfaces for stand‐in and stand‐off situations, respectively. This shows that there should be careful consideration of variations within the field. Furthermore, larger field sizes may a highly variant surface where both stand‐in and stand‐off occur in different parts of the field, yielding a highly variant field when coupled with the loss of dose at the periphery on flat surfaces.

Overall, the results highlight that there is a need for more careful and informed decisions for planning of treatments for superficial units. This study describes the steps for producing flat surface measurements and modeling simple scenarios of patient stand‐in and stand‐off using a GNU Octave script. The information garnished from the flat surface measurements highlights the importance of quantifying two‐dimensional dose distributions during planning of treatments.

## 4 CONCLUSION

Isodose plots and dose distribution maps were produced for a flat surface using seven applicator sizes and two superficial beam energies. For flat surfaces, increasing field size decreases the SA90. Some potential practice adjustments have been described, such as increasing the delivered MUs or using larger applicators to alleviate the issue of dose falloff at the periphery of a field. Utilization of the inverse square law to predict dose distribution in a superficial field for stand‐in and stand‐off was verified by comparing computed dose to dose measured using a simple wax block to simulate stand‐in and stand‐off. A GNU Octave script was written that applies an ISL correction for stand‐in or stand‐off situations across the dataset of a flat surface in the shape of a Gaussian of adjustable height and thickness. The methodology outlined in this study could be followed to allow users to produce baseline flat surface films and simulate patient stand‐in and stand‐off using simple Gaussian shapes of varying height and thickness. The study highlights the need for more extensive knowledge of superficial field dose distribution than is currently employed in clinical settings. Acquisition of this knowledge would allow clinicians and physicists to make more informed decisions during planning of patient treatments.

## CONFLICTS OF INTEREST

There are no conflict of interest to report.

## References

[1] Khan FM . The physics of radiation therapy, 4th edn Baltimore and Philadelphia: Lippincott Williams & Wilkins; 2010.

[2] Allisy‐Roberts P , Williams J . Farr’s physics for medical imaging, 2nd edn Croydon, UK: Elsevier Limited; 2008.

[3] Gerig L , Soubra M , Salhani D . Beam characteristics of the Therapax DXT300 orthovoltage therapy unit. Phys Med Biol. 1994;39:1377–1392. 15552111 10.1088/0031-9155/39/9/006

[4] Gräfe J , Poirier Y , Jacso F , Khan R , Liu H , Villarreal‐Barajas JE . Assessing the deviation from the inverse square law for orthovoltage beams with closed‐ended applicators. J Appl Clin Med Phys. 2014;15(4):356–366. 10.1120/jacmp.v15i4.4893 PMC5875524 25207421

[5] Li XA , Salhani D , Ma C‐M . Characteristics of orthovoltage x‐ray therapy beams at extended SSD for applicators with end plates. J Appl Clin Med Phys. 1997;42:357–370. 10.1088/0031-9155/42/2/008 9044418

[6] Aspradakis MM , Zucchetti P . Acceptance, commissioning and clinical use of the WOmed T‐200 kilovoltage X‐ray therapy unit. Br J Radiol. 2015;88(1055):20150001. 26224430 10.1259/bjr.20150001 PMC4743444

[7] Evans PA , Moloney AJ , Mountford P . Performance assessment of the Gulmay D3300 kilovoltage X‐ray therapy unit. Br J Radiol. 2001;74(882):537–547. 11459733 10.1259/bjr.74.882.740537

[8] Palmer AL , Jafari SM , Mone I , Muscat S . Evaluation and clinical implementation of in vivo dosimetry for kV radiotherapy using radiochromic film and micro‐silica bead thermoluminescent detectors. Phys Med. 2017;42:47–54. 29173920 10.1016/j.ejmp.2017.08.009

[9] International Commission on Radiation Units and Measurements . (1976). Report 24: determination of absorbed dose in a patient irradiated by beams of X or gamma rays in radiotherapy procedures. 10.1093/jicru/os13.1.Report24.

[10] Ashland . FilmQA Pro Users Guide. http://www.gafchromic.com/filmqa-software/filmqapro/index.asp. Accessed October 3, 2018.

[11] Prentou E , Papagiannis P , Pantelis E , Zoros E , Karaiskos P . [OA153] EBT3 radiochromic film dosimetry in kV X‐ray radiation therapy. Phys Med. 2018;52(1):58–59.

[12] Villarreal‐Barajas JE , Khan R . Energy response of EBT3 radiochromic films: implications for dosimetry in kilovoltage range. J Appl Clin Med Phys. 2014;15(1):331–338. 10.1120/jacmp.v15i1.4439 PMC5711253 24423839

[13] Gill S , Hill R . A study on the use of GafchromicTM EBT3 film for output factor measurements in kilovoltage X‐ray beams. Australas Phys Eng Sci Med. 2013;36(4):465–471. 24264224 10.1007/s13246-013-0226-9

[14] Steenbeke F , Gevaert T , Tournel K . Quality Assurance of a 50‐kV radiotherapy unit using EBT3 GafChromic Film: a feasibility study. Technol Cancer Res Treat. 2016;15(1):163–170. 25575576 10.1177/1533034614565910

[15] Chiu‐Tsao SC , Ho Y , Shankar R , Wang L , Harrison LB . Energy dependence of response of new high sensitivity radiochromic films for megavoltage and kilovoltage radiation energies. Med Phys. 2005;32:3350–3354. 16370422 10.1118/1.2065467

[16] Arjomandy B , Tailor R , Anand A , et al. Energy dependence and dose response of Gafchromic EBT2 film over a wide range of photon, electron and proton beam energies. Med Phys. 2016;37(5):1942–1947. 10.1118/1.3373523 20527528

